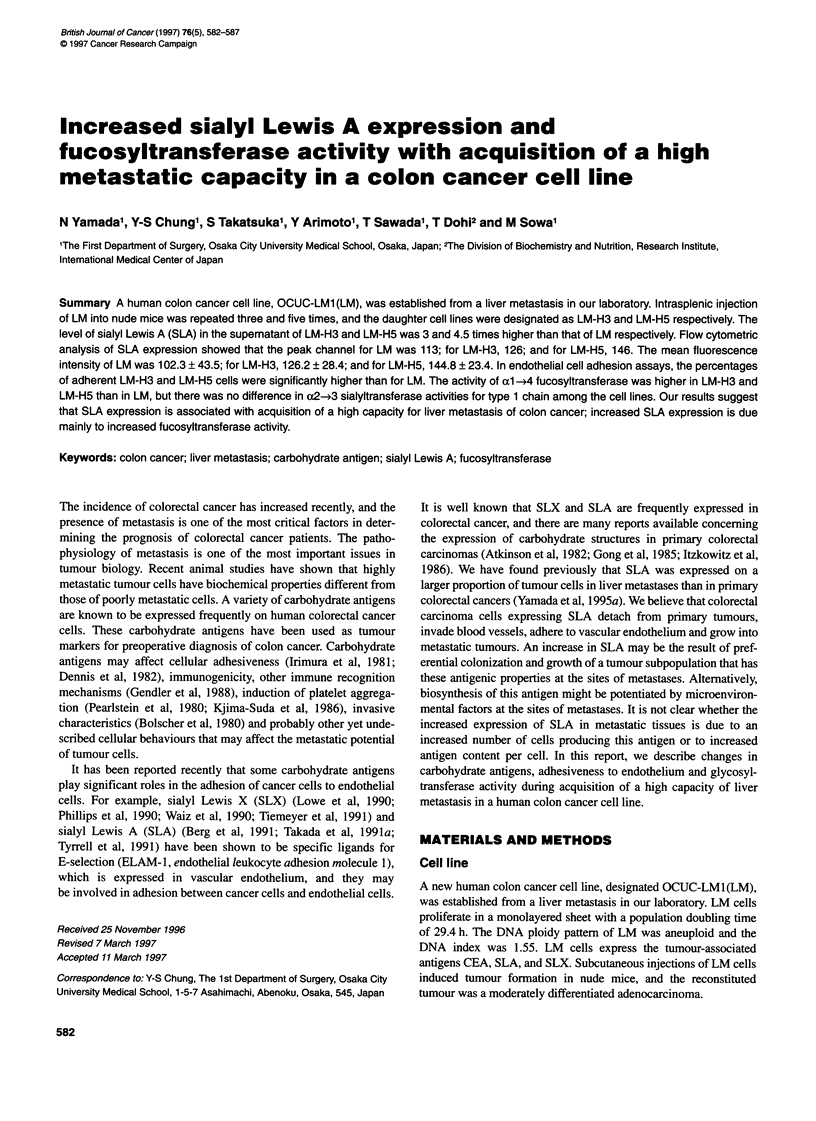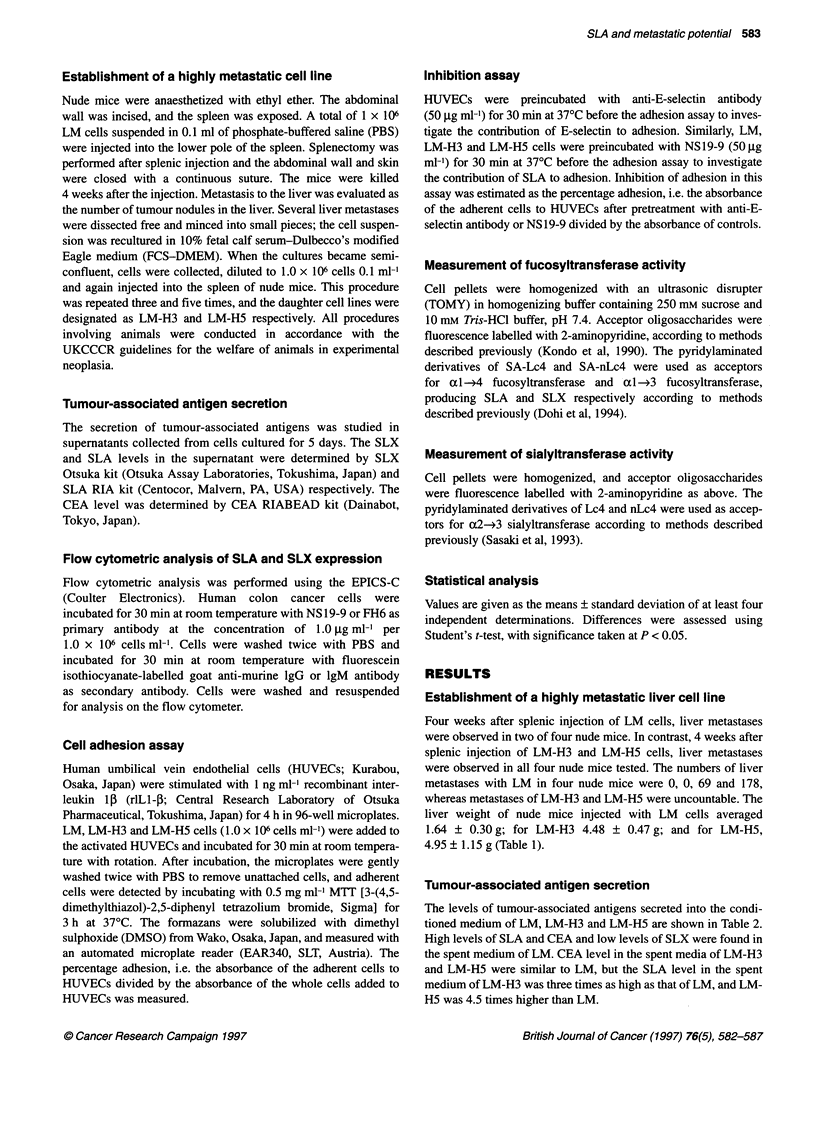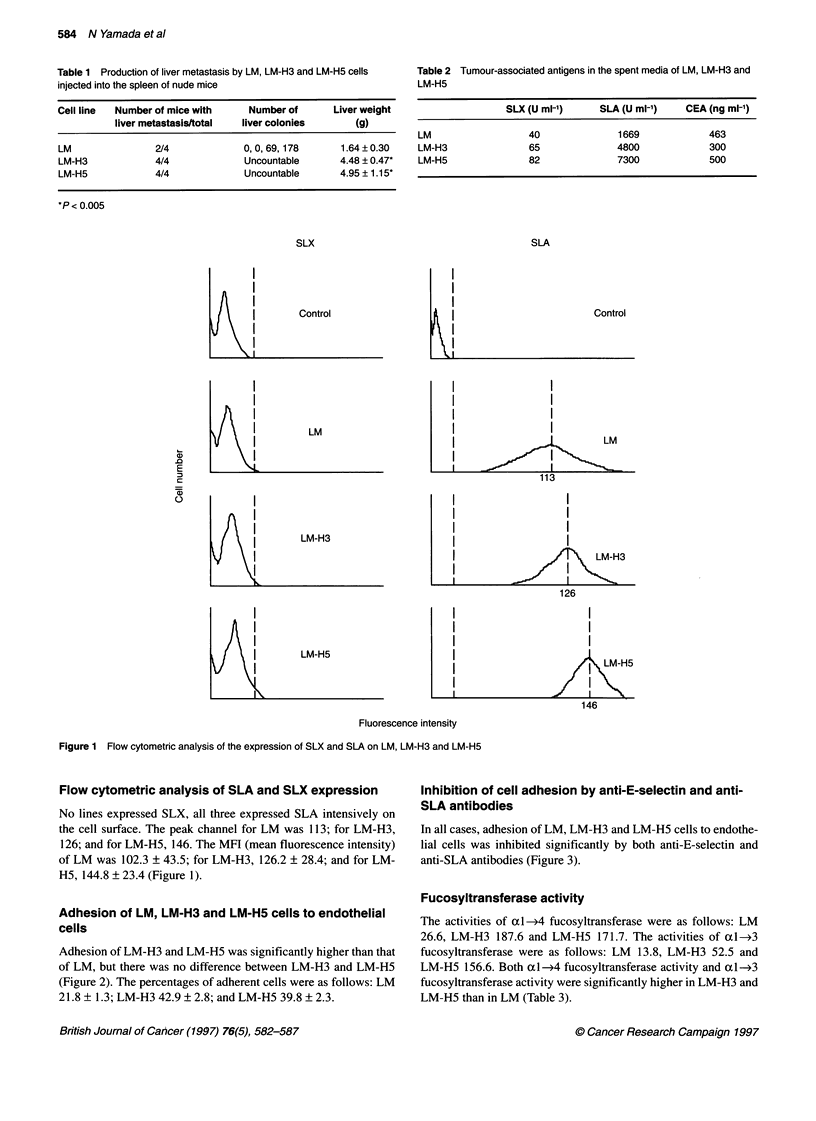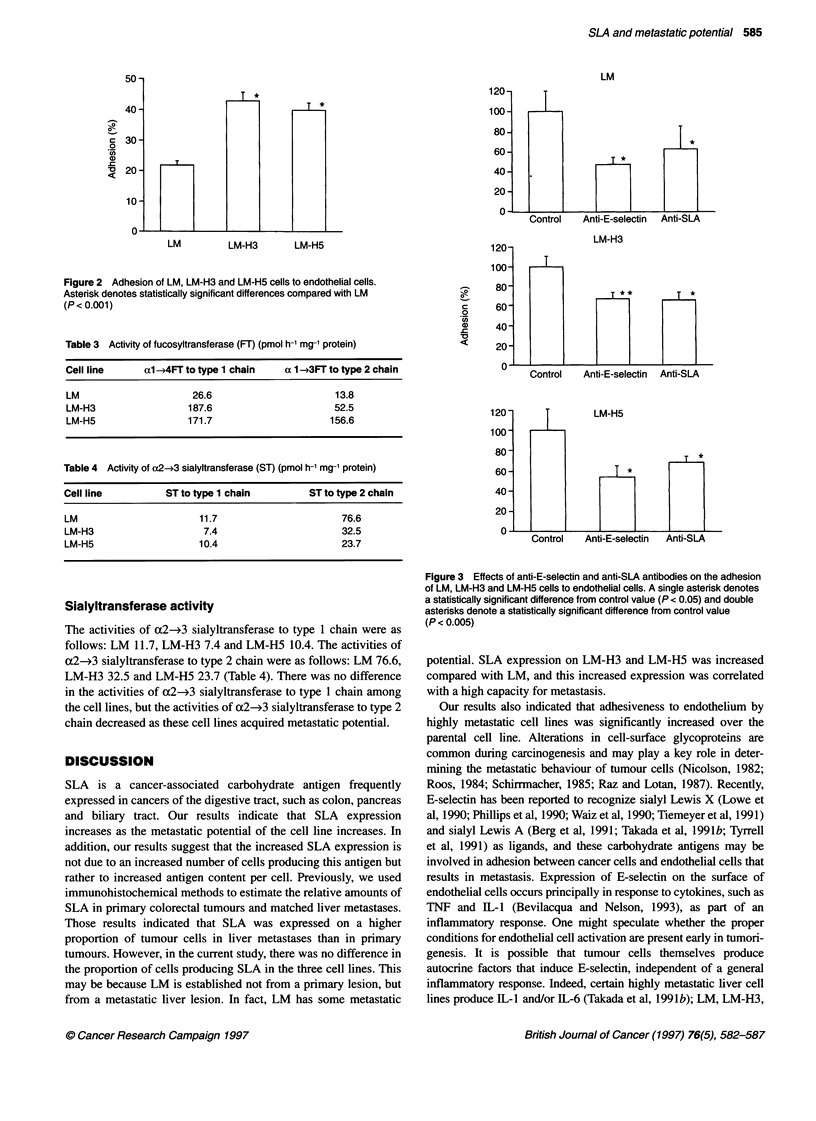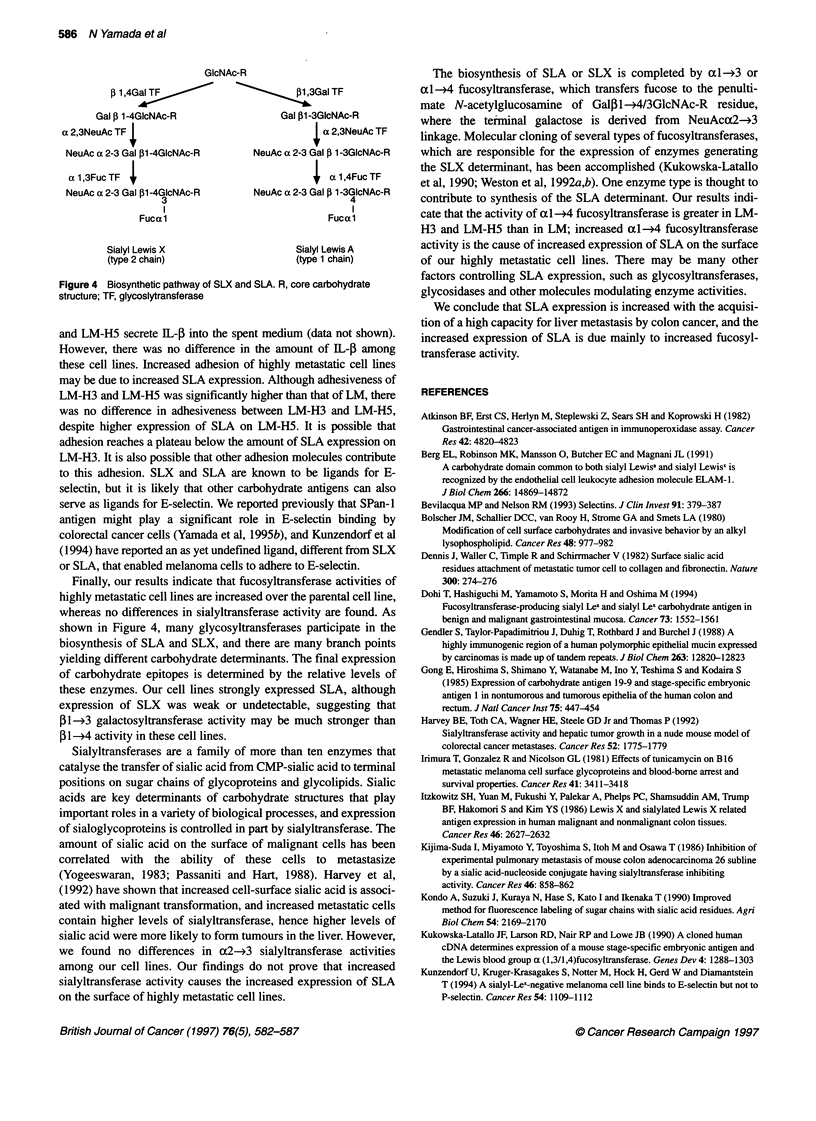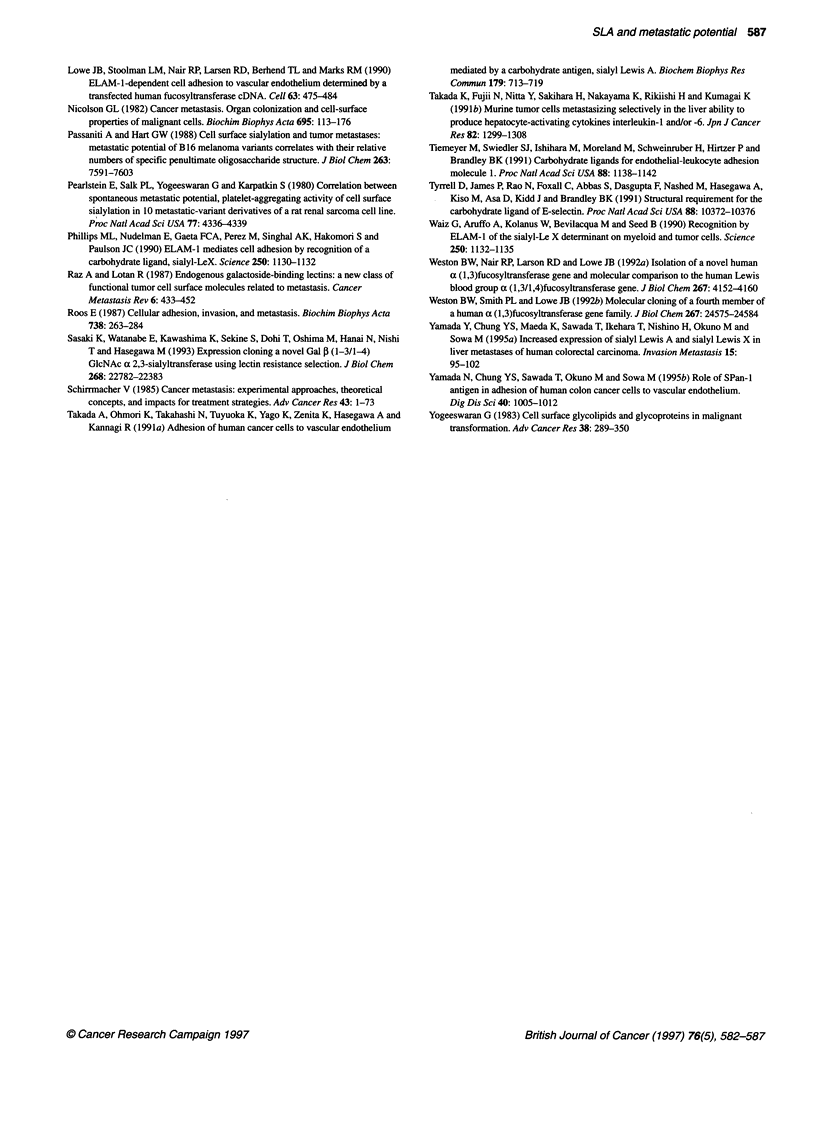# Increased sialyl Lewis A expression and fucosyltransferase activity with acquisition of a high metastatic capacity in a colon cancer cell line.

**DOI:** 10.1038/bjc.1997.429

**Published:** 1997

**Authors:** N. Yamada, Y. S. Chung, S. Takatsuka, Y. Arimoto, T. Sawada, T. Dohi, M. Sowa

**Affiliations:** The First Department of Surgery, Osaka City University Medical School, Osaka, Japan.

## Abstract

A human colon cancer cell line, OCUC-LM1(LM), was established from a liver metastasis in our laboratory. Intrasplenic injection of LM into nude mice was repeated three and five times, and the daughter cell lines were designated as LM-H3 and LM-H5 respectively. The level of sialyl Lewis A (SLA) in the supernatant of LM-H3 and LM-H5 was 3 and 4.5 times higher than that of LM respectively. Flow cytometric analysis of SLA expression showed that the peak channel for LM was 113; for LM-H3, 126; and for LM-H5, 146. The mean fluorescence intensity of LM was 102.3 +/- 43.5; for LM-H3, 126.2 +/- 28.4; and for LM-H5, 144.8 +/- 23.4. In endothelial cell adhesion assays, the percentages of adherent LM-H3 and LM-H5 cells were significantly higher than for LM. The activity of alpha1-->4 fucosyltransferase was higher in LM-H3 and LM-H5 than in LM, but there was no difference in alpha2-->3 sialyltransferase activities for type 1 chain among the cell lines. Our results suggest that SLA expression is associated with acquisition of a high capacity for liver metastasis of colon cancer; increased SLA expression is due mainly to increased fucosyltransferase activity.


British Journal of Cancer (1997) 76(5), 582–587
© 1997 Cancer Research Campaign

### Increased sialyl Lewis A expression and fucosyltransferase activity with acquisition of a high metastatic capacity in a colon cancer cell line

**N Yamada[1], Y-S Chung[1], S Takatsuka[1], Y Arimoto[1], T Sawada[1], T Dohi[2] and M Sowa[1]**

[1]The First Department of Surgery, Osaka City University Medical School, Osaka, Japan; [2]The Division of Biochemistry and Nutrition, Research Institute, International Medical Center of Japan

**Summary** A human colon cancer cell line, OCUC-LM1(LM), was established from a liver metastasis in our laboratory. Intrasplenic injection of LM into nude mice was repeated three and five times, and the daughter cell lines were designated as LM-H3 and LM-H5 respectively. The level of sialyl Lewis A (SLA) in the supernatant of LM-H3 and LM-H5 was 3 and 4.5 times higher than that of LM respectively. Flow cytometric analysis of SLA expression showed that the peak channel for LM was 113; for LM-H3, 126; and for LM-H5, 146. The mean fluorescence intensity of LM was 102.3 ± 43.5; for LM-H3, 126.2 ± 28.4; and for LM-H5, 144.8 ± 23.4. In endothelial cell adhesion assays, the percentages of adherent LM-H3 and LM-H5 cells were significantly higher than for LM. The activity of $\alpha1\rightarrow4$ fucosyltransferase was higher in LM-H3 and LM-H5 than in LM, but there was no difference in $\alpha2\rightarrow3$ sialyltransferase activities for type 1 chain among the cell lines. Our results suggest that SLA expression is associated with acquisition of a high capacity for liver metastasis of colon cancer; increased SLA expression is due mainly to increased fucosyltransferase activity.

**Keywords:** colon cancer; liver metastasis; carbohydrate antigen; sialyl Lewis A; fucosyltransferase

The incidence of colorectal cancer has increased recently, and the presence of metastasis is one of the most critical factors in determining the prognosis of colorectal cancer patients. The pathophysiology of metastasis is one of the most important issues in tumour biology. Recent animal studies have shown that highly metastatic tumour cells have biochemical properties different from those of poorly metastatic cells. A variety of carbohydrate antigens are known to be expressed frequently on human colorectal cancer cells. These carbohydrate antigens have been used as tumour markers for preoperative diagnosis of colon cancer. Carbohydrate antigens may affect cellular adhesiveness (Irimura et al, 1981; Dennis et al, 1982), immunogenicity, other immune recognition mechanisms (Gendler et al, 1988), induction of platelet aggregation (Pearlstein et al, 1980; Kjima-Suda et al, 1986), invasive characteristics (Bolscher et al, 1980) and probably other yet undescribed cellular behaviours that may affect the metastatic potential of tumour cells.

It has been reported recently that some carbohydrate antigens play significant roles in the adhesion of cancer cells to endothelial cells. For example, sialyl Lewis X (SLX) (Lowe et al, 1990; Phillips et al, 1990; Waiz et al, 1990; Tiemeyer et al, 1991) and sialyl Lewis A (SLA) (Berg et al, 1991; Takada et al, 1991*a*; Tyrrell et al, 1991) have been shown to be specific ligands for E-selection (ELAM-1, *e*ndothelial *l*eukocyte *a*dhesion *m*olecule 1), which is expressed in vascular endothelium, and they may be involved in adhesion between cancer cells and endothelial cells.

*Received 25 November 1996*
*Revised 7 March 1997*
*Accepted 11 March 1997*

*Correspondence to:* Y-S Chung, The 1st Department of Surgery, Osaka City University Medical School, 1-5-7 Asahimachi, Abenoku, Osaka, 545, Japan

It is well known that SLX and SLA are frequently expressed in colorectal cancer, and there are many reports available concerning the expression of carbohydrate structures in primary colorectal carcinomas (Atkinson et al, 1982; Gong et al, 1985; Itzkowitz et al, 1986). We have found previously that SLA was expressed on a larger proportion of tumour cells in liver metastases than in primary colorectal cancers (Yamada et al, 1995*a*). We believe that colorectal carcinoma cells expressing SLA detach from primary tumours, invade blood vessels, adhere to vascular endothelium and grow into metastatic tumours. An increase in SLA may be the result of preferential colonization and growth of a tumour subpopulation that has these antigenic properties at the sites of metastases. Alternatively, biosynthesis of this antigen might be potentiated by microenvironmental factors at the sites of metastases. It is not clear whether the increased expression of SLA in metastatic tissues is due to an increased number of cells producing this antigen or to increased antigen content per cell. In this report, we describe changes in carbohydrate antigens, adhesiveness to endothelium and glycosyltransferase activity during acquisition of a high capacity of liver metastasis in a human colon cancer cell line.

### MATERIALS AND METHODS

### Cell line

A new human colon cancer cell line, designated OCUC-LM1(LM), was established from a liver metastasis in our laboratory. LM cells proliferate in a monolayered sheet with a population doubling time of 29.4 h. The DNA ploidy pattern of LM was aneuploid and the DNA index was 1.55. LM cells express the tumour-associated antigens CEA, SLA, and SLX. Subcutaneous injections of LM cells induced tumour formation in nude mice, and the reconstituted tumour was a moderately differentiated adenocarcinoma.

### Establishment of a highly metastatic cell line

Nude mice were anaesthetized with ethyl ether. The abdominal wall was incised, and the spleen was exposed. A total of $1 \times 10^6$ LM cells suspended in 0.1 ml of phosphate-buffered saline (PBS) were injected into the lower pole of the spleen. Splenectomy was performed after splenic injection and the abdominal wall and skin were closed with a continuous suture. The mice were killed 4 weeks after the injection. Metastasis to the liver was evaluated as the number of tumour nodules in the liver. Several liver metastases were dissected free and minced into small pieces; the cell suspension was recultured in 10% fetal calf serum–Dulbecco's modified Eagle medium (FCS–DMEM). When the cultures became semi-confluent, cells were collected, diluted to $1.0 \times 10^6$ cells 0.1 $ml^{-1}$ and again injected into the spleen of nude mice. This procedure was repeated three and five times, and the daughter cell lines were designated as LM-H3 and LM-H5 respectively. All procedures involving animals were conducted in accordance with the UKCCCR guidelines for the welfare of animals in experimental neoplasia.

### Tumour-associated antigen secretion

The secretion of tumour-associated antigens was studied in supernatants collected from cells cultured for 5 days. The SLX and SLA levels in the supernatant were determined by SLX Otsuka kit (Otsuka Assay Laboratories, Tokushima, Japan) and SLA RIA kit (Centocor, Malvern, PA, USA) respectively. The CEA level was determined by CEA RIABEAD kit (Dainabot, Tokyo, Japan).

### Flow cytometric analysis of SLA and SLX expression

Flow cytometric analysis was performed using the EPICS-C (Coulter Electronics). Human colon cancer cells were incubated for 30 min at room temperature with NS19-9 or FH6 as primary antibody at the concentration of 1.0 $\mu g\ ml^{-1}$ per $1.0 \times 10^6$ cells $ml^{-1}$. Cells were washed twice with PBS and incubated for 30 min at room temperature with fluorescein isothiocyanate-labelled goat anti-murine lgG or lgM antibody as secondary antibody. Cells were washed and resuspended for analysis on the flow cytometer.

### Cell adhesion assay

Human umbilical vein endothelial cells (HUVECs; Kurabou, Osaka, Japan) were stimulated with 1 ng $ml^{-1}$ recombinant interleukin 1β (rlL1-β; Central Research Laboratory of Otsuka Pharmaceutical, Tokushima, Japan) for 4 h in 96-well microplates. LM, LM-H3 and LM-H5 cells ($1.0 \times 10^6$ cells $ml^{-1}$) were added to the activated HUVECs and incubated for 30 min at room temperature with rotation. After incubation, the microplates were gently washed twice with PBS to remove unattached cells, and adherent cells were detected by incubating with 0.5 mg $ml^{-1}$ MTT [3-(4,5-dimethylthiazol)-2,5-diphenyl tetrazolium bromide, Sigma] for 3 h at 37°C. The formazans were solubilized with dimethyl sulphoxide (DMSO) from Wako, Osaka, Japan, and measured with an automated microplate reader (EAR340, SLT, Austria). The percentage adhesion, i.e. the absorbance of the adherent cells to HUVECs divided by the absorbance of the whole cells added to HUVECs was measured.

### Inhibition assay

HUVECs were preincubated with anti-E-selectin antibody (50 $\mu g\ ml^{-1}$) for 30 min at 37°C before the adhesion assay to investigate the contribution of E-selectin to adhesion. Similarly, LM, LM-H3 and LM-H5 cells were preincubated with NS19-9 (50 $\mu g\ ml^{-1}$) for 30 min at 37°C before the adhesion assay to investigate the contribution of SLA to adhesion. Inhibition of adhesion in this assay was estimated as the percentage adhesion, i.e. the absorbance of the adherent cells to HUVECs after pretreatment with anti-E-selectin antibody or NS19-9 divided by the absorbance of controls.

### Measurement of fucosyltransferase activity

Cell pellets were homogenized with an ultrasonic disrupter (TOMY) in homogenizing buffer containing 250 mM sucrose and 10 mM *Tris*-HCl buffer, pH 7.4. Acceptor oligosaccharides were fluorescence labelled with 2-aminopyridine, according to methods described previously (Kondo et al, 1990). The pyridylaminated derivatives of SA-Lc4 and SA-nLc4 were used as acceptors for $\alpha 1 \rightarrow 4$ fucosyltransferase and $\alpha 1 \rightarrow 3$ fucosyltransferase, producing SLA and SLX respectively according to methods described previously (Dohi et al, 1994).

### Measurement of sialyltransferase activity

Cell pellets were homogenized, and acceptor oligosaccharides were fluorescence labelled with 2-aminopyridine as above. The pyridylaminated derivatives of Lc4 and nLc4 were used as acceptors for $\alpha 2 \rightarrow 3$ sialyltransferase according to methods described previously (Sasaki et al, 1993).

### Statistical analysis

Values are given as the means ± standard deviation of at least four independent determinations. Differences were assessed using Student's *t*-test, with significance taken at $P < 0.05$.

### RESULTS

### Establishment of a highly metastatic liver cell line

Four weeks after splenic injection of LM cells, liver metastases were observed in two of four nude mice. In contrast, 4 weeks after splenic injection of LM-H3 and LM-H5 cells, liver metastases were observed in all four nude mice tested. The numbers of liver metastases with LM in four nude mice were 0, 0, 69 and 178, whereas metastases of LM-H3 and LM-H5 were uncountable. The liver weight of nude mice injected with LM cells averaged 1.64 ± 0.30 g; for LM-H3 4.48 ± 0.47 g; and for LM-H5, 4.95 ± 1.15 g (Table 1).

### Tumour-associated antigen secretion

The levels of tumour-associated antigens secreted into the conditioned medium of LM, LM-H3 and LM-H5 are shown in Table 2. High levels of SLA and CEA and low levels of SLX were found in the spent medium of LM. CEA level in the spent media of LM-H3 and LM-H5 were similar to LM, but the SLA level in the spent medium of LM-H3 was three times as high as that of LM, and LM-H5 was 4.5 times higher than LM.

© Cancer Research Campaign 1997

Table 1 Production of liver metastasis by LM, LM-H3 and LM-H5 cells injected into the spleen of nude mice

| Cell line | Number of mice with liver metastasis/total | Number of liver colonies | Liver weight (g) |
|---|---|---|---|
| LM | 2/4 | 0, 0, 69, 178 | 1.64 ± 0.30 |
| LM-H3 | 4/4 | Uncountable | 4.48 ± 0.47* |
| LM-H5 | 4/4 | Uncountable | 4.95 ± 1.15* |

*$P < 0.005$

Table 2 Tumour-associated antigens in the spent media of LM, LM-H3 and LM-H5

| | SLX (U $ml^{-1}$) | SLA (U $ml^{-1}$) | CEA (ng $ml^{-1}$) |
|---|---|---|---|
| LM | 40 | 1669 | 463 |
| LM-H3 | 65 | 4800 | 300 |
| LM-H5 | 82 | 7300 | 500 |

Figure 1 Flow cytometric analysis of the expression of SLX and SLA on LM, LM-H3 and LM-H5

### Flow cytometric analysis of SLA and SLX expression

No lines expressed SLX, all three expressed SLA intensively on the cell surface. The peak channel for LM was 113; for LM-H3, 126; and for LM-H5, 146. The MFI (mean fluorescence intensity) of LM was 102.3 ± 43.5; for LM-H3, 126.2 ± 28.4; and for LM-H5, 144.8 ± 23.4 (Figure 1).

### Adhesion of LM, LM-H3 and LM-H5 cells to endothelial cells

Adhesion of LM-H3 and LM-H5 was significantly higher than that of LM, but there was no difference between LM-H3 and LM-H5 (Figure 2). The percentages of adherent cells were as follows: LM 21.8 ± 1.3; LM-H3 42.9 ± 2.8; and LM-H5 39.8 ± 2.3.

### Inhibition of cell adhesion by anti-E-selectin and anti-SLA antibodies

In all cases, adhesion of LM, LM-H3 and LM-H5 cells to endothelial cells was inhibited significantly by both anti-E-selectin and anti-SLA antibodies (Figure 3).

### Fucosyltransferase activity

The activities of $\alpha 1 \rightarrow 4$ fucosyltransferase were as follows: LM 26.6, LM-H3 187.6 and LM-H5 171.7. The activities of $\alpha 1 \rightarrow 3$ fucosyltransferase were as follows: LM 13.8, LM-H3 52.5 and LM-H5 156.6. Both $\alpha 1 \rightarrow 4$ fucosyltransferase activity and $\alpha 1 \rightarrow 3$ fucosyltransferase activity were significantly higher in LM-H3 and LM-H5 than in LM (Table 3).

© Cancer Research Campaign 1997

**Figure 2** Adhesion of LM, LM-H3 and LM-H5 cells to endothelial cells. Asterisk denotes statistically significant differences compared with LM ($P < 0.001$)

**Table 3** Activity of fucosyltransferase (FT) (pmol $h^{-1}$ $mg^{-1}$ protein)

| Cell line | α1→4FT to type 1 chain | α 1→3FT to type 2 chain |
|---|---|---|
| LM | 26.6 | 13.8 |
| LM-H3 | 187.6 | 52.5 |
| LM-H5 | 171.7 | 156.6 |

**Table 4** Activity of α2→3 sialyltransferase (ST) (pmol $h^{-1}$ $mg^{-1}$ protein)

| Cell line | ST to type 1 chain | ST to type 2 chain |
|---|---|---|
| LM | 11.7 | 76.6 |
| LM-H3 | 7.4 | 32.5 |
| LM-H5 | 10.4 | 23.7 |

### Sialyltransferase activity

The activities of α2→3 sialyltransferase to type 1 chain were as follows: LM 11.7, LM-H3 7.4 and LM-H5 10.4. The activities of α2→3 sialyltransferase to type 2 chain were as follows: LM 76.6, LM-H3 32.5 and LM-H5 23.7 (Table 4). There was no difference in the activities of α2→3 sialyltransferase to type 1 chain among the cell lines, but the activities of α2→3 sialyltransferase to type 2 chain decreased as these cell lines acquired metastatic potential.

### DISCUSSION

SLA is a cancer-associated carbohydrate antigen frequently expressed in cancers of the digestive tract, such as colon, pancreas and biliary tract. Our results indicate that SLA expression increases as the metastatic potential of the cell line increases. In addition, our results suggest that the increased SLA expression is not due to an increased number of cells producing this antigen but rather to increased antigen content per cell. Previously, we used immunohistochemical methods to estimate the relative amounts of SLA in primary colorectal tumours and matched liver metastases. Those results indicated that SLA was expressed on a higher proportion of tumour cells in liver metastases than in primary tumours. However, in the current study, there was no difference in the proportion of cells producing SLA in the three cell lines. This may be because LM is established not from a primary lesion, but from a metastatic liver lesion. In fact, LM has some metastatic

**Figure 3** Effects of anti-E-selectin and anti-SLA antibodies on the adhesion of LM, LM-H3 and LM-H5 cells to endothelial cells. A single asterisk denotes a statistically significant difference from control value ($P < 0.05$) and double asterisks denote a statistically significant difference from control value ($P < 0.005$)

potential. SLA expression on LM-H3 and LM-H5 was increased compared with LM, and this increased expression was correlated with a high capacity for metastasis.

Our results also indicated that adhesiveness to endothelium by highly metastatic cell lines was significantly increased over the parental cell line. Alterations in cell-surface glycoproteins are common during carcinogenesis and may play a key role in determining the metastatic behaviour of tumour cells (Nicolson, 1982; Roos, 1984; Schirrmacher, 1985; Raz and Lotan, 1987). Recently, E-selectin has been reported to recognize sialyl Lewis X (Lowe et al, 1990; Phillips et al, 1990; Waiz et al, 1990; Tiemeyer et al, 1991) and sialyl Lewis A (Berg et al, 1991; Takada et al, 1991*b*; Tyrrell et al, 1991) as ligands, and these carbohydrate antigens may be involved in adhesion between cancer cells and endothelial cells that results in metastasis. Expression of E-selectin on the surface of endothelial cells occurs principally in response to cytokines, such as TNF and IL-1 (Bevilacqua and Nelson, 1993), as part of an inflammatory response. One might speculate whether the proper conditions for endothelial cell activation are present early in tumorigenesis. It is possible that tumour cells themselves produce autocrine factors that induce E-selectin, independent of a general inflammatory response. Indeed, certain highly metastatic liver cell lines produce IL-1 and/or IL-6 (Takada et al, 1991*b*); LM, LM-H3,

© Cancer Research Campaign 1997

**Figure 4** Biosynthetic pathway of SLX and SLA. R, core carbohydrate structure; TF, glycoslytransferase and LM-H5 secrete IL-β into the spent medium (data not shown). However, there was no difference in the amount of IL-β among these cell lines. Increased adhesion of highly metastatic cell lines may be due to increased SLA expression. Although adhesiveness of LM-H3 and LM-H5 was significantly higher than that of LM, there was no difference in adhesiveness between LM-H3 and LM-H5, despite higher expression of SLA on LM-H5. It is possible that adhesion reaches a plateau below the amount of SLA expression on LM-H3. It is also possible that other adhesion molecules contribute to this adhesion. SLX and SLA are known to be ligands for E-selectin, but it is likely that other carbohydrate antigens can also serve as ligands for E-selectin. We reported previously that SPan-1 antigen might play a significant role in E-selectin binding by colorectal cancer cells (Yamada et al, 1995*b*), and Kunzendorf et al (1994) have reported an as yet undefined ligand, different from SLX or SLA, that enabled melanoma cells to adhere to E-selectin.

Finally, our results indicate that fucosyltransferase activities of highly metastatic cell lines are increased over the parental cell line, whereas no differences in sialyltransferase activity are found. As shown in Figure 4, many glycosyltransferases participate in the biosynthesis of SLA and SLX, and there are many branch points yielding different carbohydrate determinants. The final expression of carbohydrate epitopes is determined by the relative levels of these enzymes. Our cell lines strongly expressed SLA, although expression of SLX was weak or undetectable, suggesting that β1→3 galactosyltransferase activity may be much stronger than β1→4 activity in these cell lines.

Sialyltransferases are a family of more than ten enzymes that catalyse the transfer of sialic acid from CMP-sialic acid to terminal positions on sugar chains of glycoproteins and glycolipids. Sialic acids are key determinants of carbohydrate structures that play important roles in a variety of biological processes, and expression of sialoglycoproteins is controlled in part by sialyltransferase. The amount of sialic acid on the surface of malignant cells has been correlated with the ability of these cells to metastasize (Yogeeswaran, 1983; Passaniti and Hart, 1988). Harvey et al, (1992) have shown that increased cell-surface sialic acid is associated with malignant transformation, and increased metastatic cells contain higher levels of sialyltransferase, hence higher levels of sialic acid were more likely to form tumours in the liver. However, we found no differences in α2→3 sialyltransferase activities among our cell lines. Our findings do not prove that increased sialyltransferase activity causes the increased expression of SLA on the surface of highly metastatic cell lines.

The biosynthesis of SLA or SLX is completed by α1→3 or α1→4 fucosyltransferase, which transfers fucose to the penultimate *N*-acetylglucosamine of Galβ1→4/3GlcNAc-R residue, where the terminal galactose is derived from NeuAcα2→3 linkage. Molecular cloning of several types of fucosyltransferases, which are responsible for the expression of enzymes generating the SLX determinant, has been accomplished (Kukowska-Latallo et al, 1990; Weston et al, 1992*a*,*b*). One enzyme type is thought to contribute to synthesis of the SLA determinant. Our results indicate that the activity of α1→4 fucosyltransferase is greater in LM-H3 and LM-H5 than in LM; increased α1→4 fucosyltransferase activity is the cause of increased expression of SLA on the surface of our highly metastatic cell lines. There may be many other factors controlling SLA expression, such as glycosyltransferases, glycosidases and other molecules modulating enzyme activities.

We conclude that SLA expression is increased with the acquisition of a high capacity for liver metastasis by colon cancer, and the increased expression of SLA is due mainly to increased fucosyltransferase activity.

### REFERENCES

Atkinson BF, Erst CS, Herlyn M, Steplewski Z, Sears SH and Koprowski H (1982) Gastrointestinal cancer-associated antigen in immunoperoxidase assay. *Cancer Res* **42**: 4820–4823

Berg EL, Robinson MK, Mansson O, Butcher EC and Magnani JL (1991) A carbohydrate domain common to both sialyl Lewis[a] and sialyl Lewis[x] is recognized by the endothelial cell leukocyte adhesion molecule ELAM-1. *J Biol Chem* **266**: 14869–14872

Bevilacqua MP and Nelson RM (1993) Selectins. *J Clin Invest* **91**: 379–387

Bolscher JM, Schallier DCC, van Rooy H, Strome GA and Smets LA (1980) Modification of cell surface carbohydrates and invasive behavior by an alkyl lysophospholipid. *Cancer Res* **48**: 977–982

Dennis J, Waller C, Timple R and Schirrmacher V (1982) Surface sialic acid residues attachment of metastatic tumor cell to collagen and fibronectin. *Nature* **300**: 274–276

Dohi T, Hashiguchi M, Yamamoto S, Morita H and Oshima M (1994) Fucosyltransferase-producing sialyl Le[a] and sialyl Le[x] carbohydrate antigen in benign and malignant gastrointestinal mucosa. *Cancer* **73**: 1552–1561

Gendler S, Taylor-Papadimitriou J, Duhig T, Rothbard J and Burchel J (1988) A highly immunogenic region of a human polymorphic epithelial mucin expressed by carcinomas is made up of tandem repeats. *J Biol Chem* **263**: 12820–12823

Gong E, Hiroshima S, Shimano Y, Watanabe M, Ino Y, Teshima S and Kodaira S (1985) Expression of carbohydrate antigen 19-9 and stage-specific embryonic antigen 1 in nontumorous and tumorous epithelia of the human colon and rectum. *J Natl Cancer Inst* **75**: 447–454

Harvey BE, Toth CA, Wagner HE, Steele GD Jr and Thomas P (1992) Sialyltransferase activity and hepatic tumor growth in a nude mouse model of colorectal cancer metastases. *Cancer Res* **52**: 1775–1779

Irimura T, Gonzalez R and Nicolson GL (1981) Effects of tunicamycin on B16 metastatic melanoma cell surface glycoproteins and blood-borne arrest and survival properties. *Cancer Res* **41**: 3411–3418

Itzkowitz SH, Yuan M, Fukushi Y, Palekar A, Phelps PC, Shamsuddin AM, Trump BF, Hakomori S and Kim YS (1986) Lewis X and sialylated Lewis X related antigen expression in human malignant and nonmalignant colon tissues. *Cancer Res* **46**: 2627–2632

Kijima-Suda I, Miyamoto Y, Toyoshima S, Itoh M and Osawa T (1986) Inhibition of experimental pulmonary metastasis of mouse colon adenocarcinoma 26 subline by a sialic acid-nucleoside conjugate having sialyltransferase inhibiting activity. *Cancer Res* **46**: 858–862

Kondo A, Suzuki J, Kuraya N, Hase S, Kato I and Ikenaka T (1990) Improved method for fluorescence labeling of sugar chains with sialic acid residues. *Agri Biol Chem* **54**: 2169–2170

Kukowska-Latallo JF, Larson RD, Nair RP and Lowe JB (1990) A cloned human cDNA determines expression of a mouse stage-specific embryonic antigen and the Lewis blood group α (1,3/1,4)fucosyltransferase. *Genes Dev* **4**: 1288–1303

Kunzendorf U, Kruger-Krasagakes S, Notter M, Hock H, Gerd W and Diamantstein T (1994) A sialyl-Le[x]-negative melanoma cell line binds to E-selectin but not to P-selectin. *Cancer Res* **54**: 1109–1112

© Cancer Research Campaign 1997

Lowe JB, Stoolman LM, Nair RP, Larsen RD, Berhend TL and Marks RM (1990) ELAM-1-dependent cell adhesion to vascular endothelium determined by a transfected human fucosyltransferase cDNA. *Cell* **63**: 475–484

Nicolson GL (1982) Cancer metastasis. Organ colonization and cell-surface properties of malignant cells. *Biochim Biophys Acta* **695**: 113–176

Passaniti A and Hart GW (1988) Cell surface sialylation and tumor metastases: metastatic potential of B16 melanoma variants correlates with their relative numbers of specific penultimate oligosaccharide structure. *J Biol Chem* **263**: 7591–7603

Pearlstein E, Salk PL, Yogeeswaran G and Karpatkin S (1980) Correlation between spontaneous metastatic potential, platelet-aggregating activity of cell surface sialylation in 10 metastatic-variant derivatives of a rat renal sarcoma cell line. *Proc Natl Acad Sci USA* **77**: 4336–4339

Phillips ML, Nudelman E, Gaeta FCA, Perez M, Singhal AK, Hakomori S and Paulson JC (1990) ELAM-1 mediates cell adhesion by recognition of a carbohydrate ligand, sialyl-LeX. *Science* **250**: 1130–1132

Raz A and Lotan R (1987) Endogenous galactoside-binding lectins: a new class of functional tumor cell surface molecules related to metastasis. *Cancer Metastasis Rev* **6**: 433–452

Roos E (1987) Cellular adhesion, invasion, and metastasis. *Biochim Biophys Acta* **738**: 263–284

Sasaki K, Watanabe E, Kawashima K, Sekine S, Dohi T, Oshima M, Hanai N, Nishi T and Hasegawa M (1993) Expression cloning a novel Gal β (1–3/1–4) GlcNAc α 2,3-sialyltransferase using lectin resistance selection. *J Biol Chem* **268**: 22782–22383

Schirrmacher V (1985) Cancer metastasis: experimental approaches, theoretical concepts, and impacts for treatment strategies. *Adv Cancer Res* **43**: 1–73

Takada A, Ohmori K, Takahashi N, Tuyuoka K, Yago K, Zenita K, Hasegawa A and Kannagi R (1991*a*) Adhesion of human cancer cells to vascular endothelium mediated by a carbohydrate antigen, sialyl Lewis A. *Biochem Biophys Res Commun* **179**: 713–719

Takada K, Fujii N, Nitta Y, Sakihara H, Nakayama K, Rikiishi H and Kumagai K (1991*b*) Murine tumor cells metastasizing selectively in the liver ability to produce hepatocyte-activating cytokines interleukin-1 and/or -6. *Jpn J Cancer Res* **82**: 1299–1308

Tiemeyer M, Swiedler SJ, Ishihara M, Moreland M, Schweinruber H, Hirtzer P and Brandley BK (1991) Carbohydrate ligands for endothelial-leukocyte adhesion molecule 1. *Proc Natl Acad Sci USA* **88**: 1138–1142

Tyrrell D, James P, Rao N, Foxall C, Abbas S, Dasgupta F, Nashed M, Hasegawa A, Kiso M, Asa D, Kidd J and Brandley BK (1991) Structural requirement for the carbohydrate ligand of E-selectin. *Proc Natl Acad Sci USA* **88**: 10372–10376

Waiz G, Aruffo A, Kolanus W, Bevilacqua M and Seed B (1990) Recognition by ELAM-1 of the sialyl-Le X determinant on myeloid and tumor cells. *Science* **250**: 1132–1135

Weston BW, Nair RP, Larson RD and Lowe JB (1992*a*) Isolation of a novel human α (1,3)fucosyltransferase gene and molecular comparison to the human Lewis blood group α (1,3/1,4)fucosyltransferase gene. *J Biol Chem* **267**: 4152–4160

Weston BW, Smith PL and Lowe JB (1992*b*) Molecular cloning of a fourth member of a human α (1,3)fucosyltransferase gene family. *J Biol Chem* **267**: 24575–24584

Yamada Y, Chung YS, Maeda K, Sawada T, Ikehara T, Nishino H, Okuno M and Sowa M (1995*a*) Increased expression of sialyl Lewis A and sialyl Lewis X in liver metastases of human colorectal carcinoma. *Invasion Metastasis* **15**: 95–102

Yamada N, Chung YS, Sawada T, Okuno M and Sowa M (1995*b*) Role of SPan-1 antigen in adhesion of human colon cancer cells to vascular endothelium. *Dig Dis Sci* **40**: 1005–1012

Yogeeswaran G (1983) Cell surface glycolipids and glycoproteins in malignant transformation. *Adv Cancer Res* **38**: 289–350

© Cancer Research Campaign 1997